# Differences in Executive Functioning Between Patients with IDH1-Mutant Oligodendroglioma and Astrocytoma Before and After Surgery

**DOI:** 10.3390/cancers18010175

**Published:** 2026-01-05

**Authors:** Maud Landers-Wouters, Bart Brouwers, Geert-Jan Rutten, Elke Butterbrod

**Affiliations:** 1 Department of Neurosurgery, Elisabeth-TweeSteden Hospital Tilburg, Hilvarenbeekse Weg 60, 5022 GC Tilburg, The Netherlandse.butterbrod@vu.nl (E.B.); 2 Department of Clinical, Neuro- and Developmental Psychology, Vrije Universiteit, Boelelaan 1105, 1081 HV Amsterdam, The Netherlands

**Keywords:** oligodendroglioma, astrocytoma, long-term cognitive functioning, neurosurgery

## Abstract

Background: IDH1-mutant oligodendroglioma and astrocytoma differ not only in growth rate but also in growth pattern. Oligodendrogliomas tend to infiltrate white matter tracts, whereas astrocytomas more often displace them. Such difference could lead to different cognitive outcomes. This study examined differences in executive functioning before and up to one year after surgery between patients with IDH1-mutant astrocytoma and oligodendroglioma. Methods: Patients with WHO grade 2–3 IDH1-mutant oligodendroglioma (1p19q-codeleted) or astrocytoma were included. Cognition was assessed preoperatively, and at 3 and 12 months postoperatively using standardized computerized and paper-and-pencil tests. Groups were compared on demographics, tumor characteristics, surgical modality, extent of resection, adjuvant treatment, and baseline cognition. Longitudinal mixed models were performed to investigate differences in performances over time for the total sample and stratified by surgical approach (awake vs. asleep). Results: 162 patients (67 oligodendroglioma, 95 astrocytoma) were included. Oligodendroglioma patients were older, with more frontal and fewer temporal tumors. Oligodendroglioma patients showed a greater impairment prevalence on a measure of inhibition before surgery. In the awake surgery group, no longitudinal differences were found between diagnoses. In the asleep surgery group, astrocytoma patients remained stable while oligodendroglioma patients declined on a measure of cognitive flexibility, with performance at 3 and 12 months significantly lower than at baseline. Conclusions: Specific aspects of executive functioning in IDH1-mutant gliomas may differ by subtype. Oligodendroglioma patients showed postoperative decline in cognitive flexibility that did not recover to baseline level, particularly in case of surgery under general anesthesia. These results highlight the potential relevance of tumor subtype and surgical approach in limiting cognitive risks after glioma surgery.

## Simple Summary

People with low-grade gliomas can live for many years, so preserving their executive functioning is crucial. Two common tumor types, oligodendroglioma and astrocytoma, grow differently in the brain and may affect important white matter tracts in different ways. We studied 162 adults with these tumors, testing their executive functions (such as inhibition and cognitive flexibility) before surgery and again 3 and 12 months afterwards. We also compared patients who had awake brain surgery with mapping of cognitive functions to those who were operated on under general anesthesia. We found that patients with oligodendroglioma were more likely to show lasting decline in cognitive flexibility, particularly when operated on asleep without mapping. These findings highlight the relevance of tumor subtype and surgical approach when dealing with infiltration of important white matter tracts in predicting and limiting cognitive risks following glioma surgery.

## 1. Introduction

Cognitive dysfunction is a common symptom in patients with lower grade gliomas (LGGs). Deficits can manifest across domains at time of diagnosis [1] and can persist for years following anti-tumor treatment [2], with executive functioning being particularly vulnerable [3,4]. Such deficits are known to significantly affect daily life [5]. The degree to which surgery impacts cognitive performance remains a subject of ongoing debate. Reports indicate that up to 55% of surgically treated LGG patients experience cognitive deficits, with approximately one third of patients even showing new or worse deficits after surgery compared to their preoperative assessment [6]. At the same time, improvements following surgery are also consistently observed in glioma studies [7,8]. Patients with LGG may show less improvement following surgery than those with high grade glioma (HGG), potentially due to more pre-treatment functional reorganization and less mass effect and edema [9]. Nevertheless, deficits also seem milder in LGG in general [10]. Which patients are susceptible to decline, to remain stable or to improve post-surgery and why remains largely unclear.

There is limited research into potential differences in cognitive functioning between low grade oligodendrogliomas and astrocytomas. Whereas both tumors grow diffusely along white matter pathways, they seem to exhibit differences in growth rate and pattern [11] and may affect the functionality of white matter pathways for cognitive functioning differently [12]. In a previous investigation with MR-based tractography, we found that tumor type, as defined by 1p19q status, differently affected the Frontal Aslant Tract (FAT), with oligodendrogliomas more likely to infiltrate and astrocytomas tending to displace the tract. Clinical observations suggest that these different patterns in LGG can also be found for other major white matter pathways [13,14].

Differences in growth pattern and growth rate between IDH1-mutated oligodendrogliomas and astrocytomas may variably impact cognitive functions before and after surgery. However, as the relation between cognition and growth patterns is still unclear, it cannot consequently inform surgical planning [15]. Hypothetically, if tumor-infiltrated pathways are still functional, patients with oligodendroglioma would likely experience more pronounced (temporary) functional deterioration after resection as compared to those with astrocytoma, because tracts traversing a tumor are more likely to be damaged than pathways that are displaced by it.

The aim of the current study was to investigate potential differences in cognitive functioning, particularly focusing on executive functioning, before and up to 1 year after surgery between patients with an IDH1-mutant astrocytoma and oligodendroglioma.

## 2. Materials and Methods

### 2.1. Study Design and Patient Sample

We retrospectively analyzed data from adult patients with newly diagnosed LGGs, grade II and grade III IDH-mutated, who underwent resection between 2011 and 2023 and in whom cognitive functioning was assessed at different time points: 1–14 days before surgery, 3 months after and 12 months after surgery. Patients were included if they had undergone at least the preoperative baseline assessment. Exclusion criteria were age below 18 years, previous craniotomy, impaired testability (e.g., severe aphasia or motor dysfunction), a history of other major medical illnesses in the past year before surgery, severe neurological or psychiatric disorders in the past two years and lack of Dutch language skills. Patients did not undergo neoadjuvant treatment before surgery. A positive advice for this study was provided by the local medical ethical committee (NW2020-32, METC Brabant, Tilburg, The Netherlands). All patients included in this study provided written informed consent for usage of their data for research purposes.

### 2.2. Measures and Procedure

#### 2.2.1. Tumor Location

FLAIR images were used to assess tumor location. The following categories were distinguished: frontal, parietal, temporal, occipital and insular, whereby tumors could be located in more than one category. Assessment of tumor location was verified by at least two medical neurosurgical professionals.

#### 2.2.2. Extent of Resection

For assessment of the macroscopic extent of resection (EOR), the immediate postoperative MRI-scan was used which was conducted < 72 h after surgery. The following categories were defined: gross total (>90%), near total (>70%, <90%), subtotal (>50%, <70%) and partial (<50%). Assessment of EOR was verified by at least one neuroradiologist, neurosurgeon and neurosurgical resident.

#### 2.2.3. Adjuvant Treatment

Adjuvant treatment after surgery was assessed from electronic medical records. The following data was collected: radiotherapy within the first year, including dosage, and type of chemotherapy, including the number of cycles.

#### 2.2.4. Type of Surgery

Patients were either operated under asleep conditions without mapping or under awake conditions with intraoperative stimulation mapping if the tumor was located in or near presumed critical regions for sensorimotor or cognitive functions. This decision results from a multidisciplinary consultation that included neuro-oncologists, neuroradiologists, radiotherapists and neurosurgeons specialized in neuro-oncology. In patients that were operated under awake conditions, local anesthesia was used to allow for cortical and subcortical direct electrical stimulation (DES) of targeted cortical and subcortical areas. DES was performed with a bipolar probe (ISIS, Inomed, Emmendingen, Germany) and a biphasic current was applied (frequency 60 Hz, pulse duration 0.5–1 ms, current 3–6 mA). During cortical and subcortical stimulation as well as during resection patients performed motor and/or cognitive tasks and were continuously monitored by a trained (clinical) neuropsychologist.

#### 2.2.5. Neuropsychological Assessment

Neuropsychological screening comprised of computerized and paper-and-pencil tests was performed between 1 to 14 days prior to surgery (T0), 3 months after (T3) and 12 months after surgery (T12) and administered as part of standard clinical care. See Supplementary Table S1 for an overview and description of the included tests and cognitive domains.

The Dutch version of the computerized cognitive test battery CNS Vital Signs [16] and two commonly used pen-and-paper tasks were part of the test protocol. From the computerized battery we selected three test measures based on primary involvement of executive functioning; a shifting attention test (tapping into cognitive flexibility), and two measures of the Stroop test; specifically Stroop task 3 (tapping into response speed and inhibition of habitual responses) and the difference in reaction time on Stroop task 3–Stroop task 2 (reflecting the degree of interference patients experience in non-habitual responses vs. habitual responses). We also included a symbol digit coding test, because this test, while not traditionally viewed as an executive functional measure, is consistently predicted by cognitive flexibility alongside (psycho-)motor speed and visual scanning [17,18]. The pen-and-paper tasks included a phonemic fluency test, specifically the Dutch version of the Controlled Oral Word Association Test (COWAT) [19], and an attention and working memory test, specifically a Digit Span test with forward and backward conditions [20]. These pen-and-paper tasks were introduced to the assessment protocol as part of change in the clinical assessment protocol, and thus were available in the respective subsample of patients.

Z-scores were calculated for all test measures to adjust for age, sex and education level based on a Dutch normative control sample for the CNS Vital Signs tests, with additional correction for practice effects for follow-up measurements [16]. Z-scores for the letter fluency test were adjusted in accordance with published norms of Schmand et al. [19] and for the Digit Span test based on Schimmel et al. [21]. Scores were reversed for reaction time measures so that higher z-scores indicated better performance for all test measures.

### 2.3. Statistical Analyses

Statistical analyses were performed using Rstudio (R v4.5.1). For all analyses, we adopted significance level *α* = 0.05. Oligodendroglioma was the reference category for all analyses investigating differences between diagnoses.

#### 2.3.1. Sample Characteristics

Descriptive statistics were computed for the following participant characteristics: age at time of surgery, sex, level of education, tumor location, type of surgery (awake versus asleep), extent of resection, adjuvant treatment and baseline cognitive performance (z-scores on all tests). Diagnostic groups were compared with regard to their characteristics using independent samples t-tests or Mann-Whitney U tests (continuous variables, depending on data distribution) and Chi-square tests (categorical variables). To inspect potential bias in our follow-up sample, we statistically compared the sample characteristics and baseline test performances between patients who completed all assessments and those who did not, using a similar analysis procedure.

#### 2.3.2. Baseline Cognitive Performances

Preoperative standardized performances (z-scores) of oligodendroglioma vs. astrocytoma patients were compared univariately using independent samples t-tests or Mann–Whitney U tests (depending on data distribution). Test performances that showed a between-group difference were subsequently investigated in multiple regression analysis in which we corrected for differences in clinical characteristics.

We calculated and visualized the proportion of impaired and low performances for all tests, with impairment being defined as a performance below the 6.7th percentile (Z < −1.5) and low performances as between the 15th and 6.8th percentile of the normative group (−1.5 < Z < −1). Impairment proportions were compared between diagnoses using Chi-square tests of independence or Fisher’s exact. Measures for which impairment proportions differed were subsequently investigated using logistic regression models (dependent variable: impairment or no impairment) that corrected for differences clinical characteristics.

#### 2.3.3. Trajectories of Cognition After Surgery

We performed longitudinal mixed models (LMM) to investigate differences in the course of cognitive performances over time between diagnoses, using the nlme and lme4 package. In all models, a test measure was the dependent variable, with the three timepoints nested in patients to account for the longitudinal nature of the data. A linear effect of time was assumed across models, given the fact that included three measurements existed. We compared the fit of models with random vs. fixed slopes using the Bayesian Information Criterion. Fixed slopes were the standard, as a single uniform slope provides a simpler model. Random slopes, that allow the rate of change to vary across individuals, were adopted only if they significantly improved model fit. In order to account for the correlation between repeated measurements (i.e., the within-subject correlation), we fitted and compared the following correlation structures that hold different assumptions; autoregressive, compound symmetry and scaled identity. The structure providing the best fit for most models based on Akaike information criterion (AIC) was applied for all models.

To assess whether changes in cognitive performances over time were related to diagnosis, we included a diagnosis by time interaction in the models (oligodendroglioma as reference group). Clinical characteristics that differed between diagnostic groups were adopted as covariates. LMM were conducted in the total sample as well as stratified by surgical method (awake vs. asleep). In case of a significant interaction between time and diagnosis, we obtained the diagnosis-specific performance trajectories over time by fitting the models separately in the oligodendroglioma group and in the astrocytoma group. In addition, we compared estimated marginal means at each follow-up timepoint for the test measure, using the same covariates as the multivariable LMM. Parameters were obtained using the restricted maximum likelihood (REML) approach.

## 3. Results

### 3.1. Sample Characteristics

Sample characteristics are provided in Table 1. Groups were comparable on sociodemographic and clinical characteristics, except for age (oligodendroglioma < astrocytoma, t(160) = 4.20, *p* < 0.001), frontal lobe infiltration (oligodendroglioma > astrocytoma, *X*^2^ = 4.37, *p* < 0.05) and temporal lobe infiltration (oligodendroglioma < astrocytoma, *X*^2^ = 11.49, *p* < 0.01).

### 3.2. Pre-Surgical Functioning

Table 2 shows baseline performances (z-scores) of the complete sample and stratified by diagnosis. Independent samples-tests and Mann Whitney U tests showed that none of the baseline performances differed significantly between diagnoses, except for the Stroop test part 3 (inhibition). This difference was not statistically significant after correcting for clinical characteristics that differed between diagnoses (age, temporal lobe and frontal lobe infiltration) in the multiple regression analysis, B = 0.58, SE = 0.32, 95%CI −0.05; 1.22, *p* = 0.07. None of the clinical characteristics in the model were significantly related to the preoperative performance on this measure, *p*’s > 0.05.

**Figure 1:**
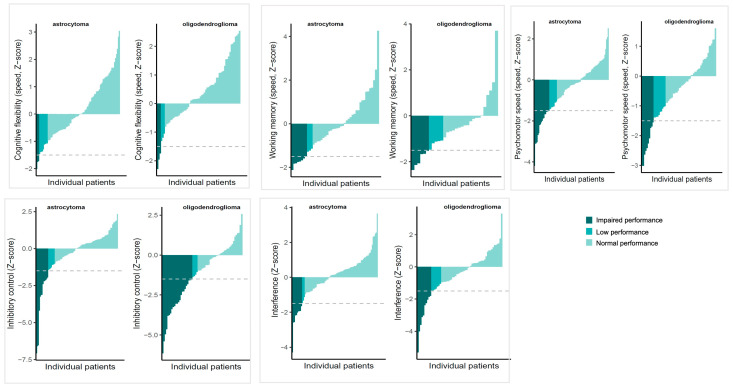
Overview of scores of individual patients ordered from lowest (left of each graph) to highest (right of each graph), stratified by diagnosis. Colours indicate clinical classifications of preoperative performances, with impaired performance: Z < −1.5, low performance: Z −1.49 to −1, normal performance: Z > −1. The grey interrupted line indicates the impairment cut-off.

Figure 1 shows performances according to the used clinical cut-offs (normal, low or impaired performance) for both diagnostic groups. The proportion of patients showing preoperative impairment was similar for oligodendroglioma patients and astrocytoma patients for all measures, except for Stroop test part 3 (38% vs. 15% respectively; *X*^2^ = 10.377, *p* = 0.01). This difference remained statistically significant after correcting for clinical characteristics that differed between diagnostic groups (age, temporal lobe and frontal lobe infiltration) in the logistic regression, B = 1.02, SE = 0.45, OR = 2.78, *p* = 0.03. The OR (odds ratio) indicates that the odds of having impairment on the Stroop test part 3 before surgery were 2.78 times higher for oligodendroglioma patients. Of the clinical characteristics in this model, frontal lobe infiltration was significantly, negatively related to impairment, B = −1.40, SE = 0.64, OR = 0.24, *p* = 0.03.

The baseline performances of the patient sample that completed follow-up vs. those who did not complete follow-up were compared in order to inspect bias in the long-term sample, but were not significantly different (all *p*’s > 0.05, Supplementary Table S2), indicating that the two groups were similar in terms of baseline cognition. We note that the mean difference between these groups on the interference measure (test 3–test 2) of the Stroop test showed a trend towards significance (incomplete follow up M = −0.35 vs. complete follow up M = 0.07, *p* = 0.054).

### 3.3. Trajectories of Cognition After Surgery in the Entire Sample

Supplementary Table S3 shows results from the linear mixed models assessing performances across the measurements (baseline, 3- and 12-month follow-up) in the total sample. Models included the following clinical variables, as these differed significantly between diagnostic groups: age at time of surgery, frontal lobe tumor location and temporal lobe tumor location.

**Figure 2:**
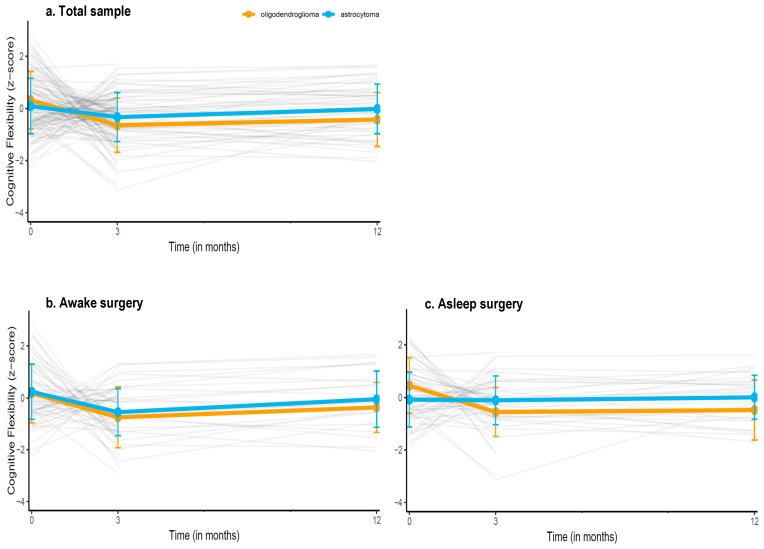
Descriptive trajectories of patients’ mean performances over time on the Shifting Attention test (measuring cognitive flexibility), in (**a**) the entire sample, (**b**) the sample undergoing awake surgery, and (**c**) the sample undergoing asleep surgery. Grey lines indicate individual patients z-scores over time, the orange and blue line indicate the mean group performance (±SDs) of oligodendroglioma and astrocytoma patients respectively. Estimated performance trajectories based on the multivariable models are provided in the Supplementary Materials.

Figure 2 shows the performances of patients over time (individual trajectories and group means) and Supplementary Figure S1 shows the estimated performance trajectory based on the model (i.e., estimated performances of diagnostic groups while accounting for the clinical variables in the model). We found a significant, positive interaction between time and astrocytoma diagnosis for the Shifting Attention test (B = 0.34, 95%CI 0.02–0.67, *p* = 0.04), indicating that patients with astrocytoma had a better trajectory for this test compared to oligodendroglioma patients. Specifically, with each next measurement, astrocytoma patients did 0.34 SD better than oligodendroglioma patients. The estimated marginal means based of the multivariable models for the Shifting Attention test at the 3 month (oligodendroglioma: −0.37 ± 0.17 astrocytoma: −0.36 ± 0.12) and the 12 month measurement (oligodendroglioma: 0.42 ± 0.22, astrocytoma: −0.12 ± 0.14) were not statistically significant, *p* > 0.05.

There were no differences in any of the other test performances over time between diagnoses. Among clinical variables, age was negatively associated with performance on the Stroop test part 3 (B = −0.02, 95%CI = −0.05; −0.002, *p* = 0.04), indicating that with increasing age, the overall performance on this test was worse. Time was negative associated with performance on the Stroop test part 3 (B = −0.45, 95%CI = −0.79; −0.13, *p* = 0.01), meaning that patients’ average performance declined over time, irrespective of other characteristics. Neither frontal nor temporal lobe infiltration were related to cognitive performances, *p*’s > 0.05.

### 3.4. Trajectories of Cognition After Awake and Asleep Surgery

Supplementary Table S4 shows the results from the stratified models. There was a significant positive interaction between time and astrocytoma diagnosis for the Shifting Attention test in the asleep surgery group (B = 0.52, 95%CI = 0.06; 0.97, *p* = 0.02), but not in the awake surgery group (B = 0.16, 95%CI = −0.30; 0.63, *p* = 0.49), indicating that astrocytoma patients who underwent asleep surgery had a better trajectory over time than oligodendroglioma patients who underwent asleep surgery. Specifically, with each next measurement, astrocytoma patients did 0.52 SD better than oligodendroglioma patients. Upon inspection of the trajectory in each diagnostic group, we found that oligodendroglioma patients who underwent asleep surgery showed moderate, significant decline over time (B = −0.55, 95%CI = −0.97; −0.14, *p* = 0.01), while astrocytoma patients remained stable (B = −0.01, 95% CI = −0.27; 0.25, *p* > 0.05).

By adopting time as a factor variable (thereby comparing each follow-up to the preoperative baseline as reference), we observed that oligodendroglioma patients performed significantly worse on the Shifting attention test at both their 3 (B = −0.80, 95%CI = −1.24; −0.37, *p* < 0.01) and 12 month follow-up (B = −0.74, 95%CI = −1.34; −0.14, *p* = 0.02) compared to their own baseline, indicating that performance did not recover to the preoperative level. Astrocytoma patients performed worse at their 3 month (B = −0.45, 95% CI = −0.76; −0.15), but not their 12 month follow-up (B = −0.15, 95% CI = −0.53; 0.23) compared to their baseline, suggesting recovery on the longer term.

Diagnosis was not related to any of the cognitive test performances over time in the awake surgery group.

## 4. Discussion

This study investigated potential differences in the trajectory of performances of executive functioning between patients with IDH1-mutant oligodendroglioma and astrocytoma up to one year after surgery. At pre-operative baseline, performances were largely comparable between groups, except for a measure of inhibition. Overall, both groups demonstrated largely similar and mostly stable trajectories for most measures, with the exception of inhibition where patients showed decline over time on average, independent of diagnosis. A difference between diagnoses existed for cognitive flexibility over time (measured with a computerized Shifting Attention test), where oligodendroglioma patients, particularly those who underwent asleep surgery, demonstrated worse performance over time compared to astrocytoma patients. Notably, whereas astrocytoma patients showed recovery after temporary decline, oligodendroglioma patients performance at 12 months was worse compared to their baseline. Clinical characteristics (age, tumor location) were differentially related to some of the test performances. These findings suggest potentially meaningful differences in executive functioning between LGG subtypes.

Our preoperative results support studies that report executive functioning in lower grade glioma being relatively well preserved on average. Still, up to a quarter of patients showed clinical impairment, depending on the specific domain. Impairment was substantially less prevalent lower compared to IDH-1 wildtype glioma, in line with previous studies [10,23]. The slower-growing nature of IDH-mutant glioma has been hypothesized to allow for better functional reorganization and compensation compared to the more disruptive growth of IDH-wildtype glioma [10]. The absence of preoperative performance differences between diagnostic groups in our sample for most tests indicates that individuals with untreated IDH-1 mutated astrocytoma and oligodendroglioma do not present with vastly different levels of executive functioning. However, impairment rates on a test of inhibition specifically were more than twice as high among oligodendroglioma compared to astrocytoma patients (38% vs. 15%), irrespective of clinical characteristics. This does point toward a potential difference in vulnerability of specific executive domains of which the potential mechanisms should be further explored. Since oligodendroglioma tend to infiltrate white matter tracts, more impairment may reflect greater tumor infiltration, for example of frontal white matter tracts, such as the FAT and Frontostriatal tract (FST), a finding supported by prior intraoperative stimulation and tractography studies [13,24].

Oligodendroglioma patients also showed a worse postoperative trajectory than astrocytoma patients on cognitive flexibility, particularly if surgery was performed under general anesthesia. In fact, although both groups performed worse on their 3 month measurement compared to their own pre-surgical baseline, only oligodendroglioma patients were still performing worse than their baseline at 12 months while astrocytoma patients had recovered. Notably, when surgery was performed under awake conditions with intraoperative mapping, both groups showed similar trajectories. This finding cautiously supports the hypothesis that in oligodendroglioma patients, functional tracts infiltrated by the tumor are more like to be damaged during resection, resulting in worse (and potentially long-term) cognitive outcomes. Conversely, mostly displaced tracts in patients with astrocytoma may remain structurally and functionally intact [13], reflected in a more stable trajectory of functioning and/or potential for recovery. Subcortical mapping could help preserve functionally relevant pathways for executive functions, particularly in tumors with an infiltrative growth pattern, and be particularly important to minimize long-term cognitive decline in oligodendroglioma patients. More generally, the absence of intraoperative mapping may leave infiltrative tumors more vulnerable to subtle but lasting executive dysfunction. To our knowledge, no prior studies have directly compared LGG subtypes in this context, so findings such as these should be further explored.

Our results highlight the potential clinical importance of considering tumor subtype in presurgical planning and patient counseling. While oligodendrogliomas are often associated with favorable oncological prognosis, they may carry greater risk for preoperative impairment and postoperative decline in specific functions, independent of tumor location, particularly when intraoperative mapping or tractography is not used. In daily life, a deterioration in functions like cognitive flexibility can manifest as having more trouble in situations that require multitasking. Given the median age at diagnosis of IDH-1 mutated glioma of about 40 to 45 years [25], it is likely that such cognitively demanding situations occur often and in different settings (e.g., at home, in social settings and at work). At the same time, cognitive test performance does not necessarily translate to a particular level of functioning in daily life [26,27], and in-clinic neuropsychological assessment is limited in its explanation of everyday tasks [28]. Furthermore, we note that the models provide average effects, and cognitive outcomes for individual patients remain difficult to predict in a reliable manner [29]. As has been shown before in glioma patients, varying individual trajectories are likely masked in group-level results [30]. Adequate monitoring of both cognition and daily functioning of glioma patients can help to identify those patients who do show dysfunction or decline that actually disrupts daily activities. Finally, our results also indicate that characteristics such as age can still be of importance, even within a relatively young patient group.

A key strength of this study is the inclusion of a relatively large and molecularly well-characterized sample, with both short- and long-term longitudinal follow-up and solid correction for phenomena that can create substantial error in repeated cognition measurements, such as test-retest reliability and practice effects. The combined use of computerized and classical neuropsychological measures enhances ecological validity, and aligns with movements towards hybrid care. However, several limitations should be acknowledged. First, the observational and retrospective nature of the study limits causal inference. The lack of postoperative tractography limits the degree to which we can conclude the actual cause of different cognitive outcome, although differences in clinical characteristics as investigated here did not appear to be meaningful drivers. Related to this, it remains difficult to conclude why group differences were observed only for the Shifting Attention task and not for other executive function measures. This also limits the generalizability of our conclusions regarding executive functioning more broadly. One possible explanation is that the Shifting Attention task, because of the flexibility requirement in combination with time pressure, may be particularly sensitive to disruption of specific frontal white matter pathways—such as the FAT or frontostriatal tracts. This hypothesis requires further studying alongside other (clinical) factors that may still be of importance. Finally, baseline characteristics and baseline cognition were largely comparable between patients who completed follow-up measurements vs. those who did not. Still, losing a portion of our sample at the twelve-moth follow-up may have introduced some attrition bias. Because reasons for drop-out and the clinical status were unknown at time of attrition we cannot conclude whether e.g., early progression has biased the long-term results, although unlikely in a 12 month follow-up in IDH-1 mutated glioma.

This study is a first exploration of the degree to which IDH-1 mutated glioma subtypes can show different cognitive outcomes. Future studies should incorporate postoperative MR tractography to directly assess structural integrity of white matter tracts post-surgery in order to evaluate whether any observed cognitive decline—particularly in oligodendroglioma patients—follows structural disruption of the infiltrated pathways. Individual-level cognitive change, for example using regression-based reliable change indices, can be highly useful to be related to MR tractography in order to assess to what degree decline on an individual level was related to structural integrity. By correlating tract damage with cognitive outcomes, such studies could clarify whether functional deterioration is driven by resection-related injury to still-functional, tumor-infiltrated tracts. In addition, integrating intraoperative stimulation data with postoperative imaging could further delineate structure–function relationships across tumor subtypes. Prospective, multimodal designs combining cognitive monitoring, advanced imaging, and surgical mapping would enhance our understanding of the neurocognitive consequences of different LGG growth patterns. Finally, the development of surgical planning tools could help mitigate cognitive risks in vulnerable subgroups.

## 5. Conclusions

Our findings suggest that executive functioning in patients with IDH1-mutant low-grade gliomas may differ based on tumor subtype. Impairment in inhibitory function was more prevalent among oligodendroglioma patients than among astrocytoma patients before surgery. Oligodendroglioma patients, particularly those operated without intraoperative mapping, showed more decline in cognitive flexibility postoperatively, with performances still significantly worse at 12-month follow-up compared to their baseline. These results highlight the relevance of tumor subtype and, potentially, choice of surgical approach when dealing with infiltration of important white matter tracts in predicting and limiting cognitive risks following glioma surgery.

## Figures and Tables

**Table 1:** Characteristics of the patient sample.

Characteristic	Oligodendroglioma (1p-19q Codeletion)	Astrocytoma	Total Sample
	**(N = 67)**	**(N = 95)**	**(N = 162)**
Age (med, range)	48.0 [20.0, 71.0] *	37.0 [19.0, 73.0] *	41.5 [19.0, 73.0]
Male	38 (56.7%)	60 (63.2%)	98 (60.5%)
Educational level ^†^			
Vocational	14 (20.9%)	16 (16.8%)	30 (18.5%)
Intermediate	26 (38.8%)	30 (31.6%)	56 (34.6%)
Higher	27 (40.3%)	49 (51.6%)	76 (46.9%)
Infiltration			
Right hemisphere	36 (53.7%)	52 (54.7%)	88 (54.3%)
Left hemisphere	32 (47.8%)	46 (48.4%)	78 (48.1%)
Frontal lobe	55 (82.1%) *	64 (67.4%) *	119 (73.5%)
Temporal lobe	10 (14.9%) *	38 (40.0%) *	48 (29.6%)
Insula	6 (9.0%)	16 (16.8%)	22 (13.6%)
Parietal lobe	10 (14.9%)	26 (27.4%)	36 (22.2%)
Occipital lobe	2 (3.0%)	3 (3.2%)	5 (3.1%)
Histopathological grade			
II	55 (82.1%)	76 (80.0%)	131 (80.9%)
III	12 (17.9%)	19 (20.0%)	31 (19.1%)
Volume (mm^3^)	34,530 ± 30,012	39,285 ± 43,826	37,738 ± 37,997
Awake surgery	30 (44.8%)	51 (53.7%)	81 (50.0%)
Macroscopic EOR			
gross total (>90%)	9 (13.4%)	20 (21.1%)	29 (17.9%)
near total (70–90%)	19 (28.4%)	37 (38.9%)	56 (34.6%)
subtotal (50–70%)	27 (40.3%)	24 (25.3%)	51 (31.5%)
partial (<50%)	12 (17.9%)	14 (14.7%)	26 (16.0%)
Adjuvant treatment			
None (before T12)	34 (51%)	47 (50%)	81 (50%)
Protontherapy + chemotherapy	10 (15%)	18 (19%)	28 (17%)
Radiotherapy + chemotherapy	18 (27%)	27 (28%)	45 (28%)
Chemotherapy only	0 (0%)	1 (1%)	1 (1%)
Radiotherapy, NOA	5 (7.5%)	1 (1.1%)	6 (3.7%)
Total dosage received (Gy)	21.8 (26.9)	22.8 (26.8)	22.4 (26.7)

* significant difference between diagnostic groups, *p* < 0.05. ^†^ Educational level according to Verhage system [22] was translated into three groups: elementary school up to lower vocational education (generally 10–13 years of education); intermediate vocational education (e.g., generally 10–14 years total education); preparatory scientific education, bachelor’s degree or higher (generally more than 12 years education). EOR = extent of resection, NOA = not otherwise specified. Chemotherapy: Temodal, Lomustine or PCV (Procarbazine-Lomustine-Vincristine combination).

**Table 2:** Baseline test performances.

Test at Baseline	Oligodendroglioma (1p-19q Codeletion)	Astrocytoma	Total Sample
Symbol Digit Coding (*n* = 150) M (sd)	−0.53 (1.07)	−0.45 (1.24)	−0.49 (1.17)
N (%) impaired	10 (16%)	18 (20%)	28 (19%)
Shifting attention test (*n* = 150) M (sd)	0.32 (1.10)	0.12 (1.10)	0.20 (1.10)
N (%) impaired	3 (4.8%)	3 (3.4%)	6 (4%)
Stroop 3 (*n* = 149) M (sd)	−1.16 (1.86) *	−0.40 (1.71) *	−0.70 (1.81)
N (%) impaired	23 (38%) *	13 (15%) *	36 (24%)
Stroop interference (*n* = 148) M (sd)	−0.50 (1.48)	0.03 (1.26)	−0.19 (1.38)
N (%) impaired	10 (16%)	10 (11%)	20 (14%)
Letter fluency (*n* = 122) M (sd)	−0.45 (1.22)	−0.40 (1.08)	−0.42 (1.13)
N (%) impaired	9 (18%)	12 (17%)	21 (17%)
Digit span forward (*n* = 75) M (sd)	−0.06 (0.98)	0.09 (1.00)	0.03 (0.99)
N (%) impaired	3 (10%)	3 (6.7%)	6 (8%)
Digit span backward (*n* = 75) M (sd)	−0.52 (1.21)	−0.10 (1.31)	−0.27 (1.28)
N (%) impaired	6 (20%)	8 (18%)	14 (19%)

* significant difference between diagnostic groups (univariable comparison testing), *p* < 0.05.

## Data Availability

The data has been stored in a protected institutional repository. Due to privacy reasons only meta-data and codes are available upon request.

## Supplementary Materials

The following supporting information can be downloaded at: https://www.mdpi.com/article/10.3390/cancers18010175/s1, Table S1: Overview of cognitive tests, domains, score calculations and descriptions; Table S2: Baseline performances of patients who complete vs. incomplete follow-up; Table S3: Results from the linear mixed models for all tests measures in the entire sample (not stratified); Table S4: Results from the linear mixed models for all test measures, with coefficients stratiefied according to surgery type; Figure S1: Estimated performances over time on the Shifting Attention test for the entire sample and each surgical modality. Reference [31] is cited in the supplementary materials.

## Abbreviations

FAT	frontal aslant tract
LGG	low-grade glioma
EF	executive function
FST	frontostriatal tract
HGG	high-grade glioma
EOR	extent of resection
DES	Direct Electrical Stimulation
