# Supplementary material for: Differences in Executive Functioning Between Patients with IDH1-Mutant Oligodendroglioma and Astrocytoma Before and After Surgery
Source: Cancers (Basel). 2026 Jan 5;18(1):175. doi: 10.3390/cancers18010175 (PMC12784891; doi:10.3390/cancers18010175)
Supplement: Supplementary file 1 [file cancers-18-00175-s001.zip › cancers-4028673-supplementary.pdf]

Differences in executive functioning between patients with IDH1-mutant oligodendroglioma and astrocytoma before and after surgery

Supplementary materials

Supplementary table 1

Cognitive test	Primary domain	Score calculation	Description
Symbol digit coding^	Psychomotor speed<br>Cognitive flexibility	Correct responses	Number 1 to 9 refer to different symbols. In 90 seconds as many numbers as possible have to be matched to the symbols
Shifting attention test^	Cognitive flexibility<br>Inhibition	Correct matches<br>False matches	Geometric objects are presented for 2 minutes and tasks switches to match them either by shape or color.
Stroop test part 3^	Response inhibition<br>Cognitive flexibility	Reaction time part 3	Part 3: Press spacebar if color and word meaning do not match
Stroop test, 3-2^	Interference	Reaction time part 3 - Reaction time part 2	Part 2: Press spacebar when color of the word matches what the word says
Digit span forward†	Short term memory/attention	Correct span with highest amount of numbers	Span of numbers is read out and should be verbally repeated, starting with two numbers and ends in case of two times an error in the span
Digit span backward†	Working memory	Idem	Span should be repeated in reverse order
Phonemic fluency††	Phonemic fluency<br>Cognitive flexibility<br>Inhibition<br>Working memory	Total number of correct words	Naming of as many words as possible in 60 seconds, starting with a specific letter of the alphabet. Repeated 2 times with different starting letters (D-A-T at T0 and T12, K-O-M at T3).

^ Computerized test from the CNS Vital Signs battery [32]

[†] from the Wechsler memory scale (paper and pencil test) [20]

[††] Dutch version of the Controlled Oral Word Association Test (paper and pencil test) [19].

Supplementary table 2. Baseline performances of patients with complete vs incomplete follow-up

**Test (at baseline)**	**Incomplete follow-up**	**Complete follow-up**	***p***	**95% CI (lower – upper)**	
Symbol Digit Coding M(sd)	-0.59(1.22)	-0.34(1.09)	.20	-0.63	0.13
Shifting attention test M(sd)	-0.68(1.57)	0.45(1.30)	.34	-0.71	0.25
Stroop part 3 M(sd)	-0.92 (2.00)	-0.40(1.46)	.08	-1.12	0.06
Stroop interference M(sd)	-0.37 (1.50)	0.07(1.14)	.054	-0.89	0.01
Letter fluency M(sd)	-0.40 (1.10)	-0.40(1.18)	.90	-0.44	0.38
Digit span forward M(sd)	0.04 (0.99)	0.04(1.00)	.97	-0.47	0.45
Digit span backward M(sd)	-0.43 (0.98)	-0.12(1.51)	.97	-0.91	0.28

Supplementary table 3. Results from the linear mixed models for all test measures in the entire sample (not stratified)

		**Symbol digit Coding***	**Shifting Attention***	**Stroop – interference***	**Stroop – part 3***	**Letter fluency**	**Digit span Forward**	**Digit span Backward**
Time x Diagnosis astrocytoma (vs oligodendroglioma)	B (SE)	0.13 (0.14)	**0.24 (0.17)**	-0.21 (0.20)	0.19 (0.20)	0.01 (0.11)	0.13 (0.14)	-0.03 (0.11)
	p	.38	**.04**	.29	.34	.91	.38	.82
	95%CI	-0.15 – 0.41	**0.01 – 0.67**	-0.61 – 0.19	-0.20 – 0.60	-0.20 – 0.23	-0.16 – 0.41	-0.26 – 0.21
Diagnosis astrocytoma (vs oligodendroglioma)	B (SE)	-0.00 (0.32)	-0.6 (0.31)	0.68 (0.39)	0.36 (0.45)	-0.01 (0.28)	-0.00 (0.31)	0.41 (0.34)
	p	.99	.05	.08	.43	.98	.99	.23
	95%CI	-0.63 – 0.63	-1.22 – 0.02	-0.09 – 1.46	-0.54 – 1.26	-0.56 – 0.54	-0.63 – 0.62	-0.26 – 1.08
Age (in years)	B (SE)	-0.00 (0.01)	-0.01 (0.01)	-0.01 (0.01)	**-0.02 (0.01)**	-0.00 (0.01)	-0.00 (0.01)	0.00 (0.01)
	p	.59	.31	.45	**.04**	.62	.59	.71
	95%CI	-0.02 – 0.01	-0.02 – 0.00	-0.02 – 0.01	-0.05 - -0.01	-0.02 – 0.01	-0.01 – 0.01	-0.01 – 0.02
Frontal location	B (SE)	-0.07 (0.21)	0.25 (0.15)	-0.08 (0.23)	-0.46 (0.35)	-0.27 (0.24)	0.07 (0.22)	0.10 (0.28)
	p	.74	.10	.74	.34	.27	.74	.73
	95%CI	-0.36 – 0.51	-0.05 – 0.56	-0.54 – 0.38	-1.15 – 0.24	-0.74 – 0.21	-0.36 – 0.50	-0.45 – 0.65
Temporal location	B (SE)	-0.13 (0.22)	0.24 (0.15)	0.01 (0.22)	-0.32 (0.34)	-0.15 (0.23)	-0.31 (0.22)	-0.08 (0.27)
	p	.55	.11	.95	.34	.52	.55	.78
	95%CI	-0.57 – 0.31	-0.05 – 0.54	-0.43 – 0.46	-1.00 – 0.35	-0.61 – 0.31	-0.57 – 0.30	-0.63 – 0.48
Time	B (SE)	-0.06 (0.08)	**-0.48 (0.13)**	0.05 (0.16)	**-0.45 (0.17)**	0.03 (0.09)	-0.02 (0.11)	-0.04 (0.09)
	p	.44	**.00**	.74	**.01**	.75	.79	.63
	95%CI	-0.22 – 0.10	**-0.74 – -0.21**	-0.27 – 0.38	**-0.79 – -0.13**	-0.15 – 0.21	-0.25 – 0.20	-0.22 – 0.14

*computerized test

Supplementary table 4. Results from the linear mixed models for all test measures, with coefficients stratified according to surgery type.

		Symbol digit Coding*	Shifting Attention*	Stroop – interference*	Stroop – part 3*	Letter fluency	Digit span Forward	Digit span Backward
**Time x Diagnosis astrocytoma (vs oligodendroglioma)**								
awake surgery	B (SE)	-0.14(0.16)	0.16(0.24)	0.03(0.28)	0.47(0.28)	0.03(0.16)	0.01(0.19)	-0.02(0.17)
	p	.41	.49	.93	.09	.86	.98	.89
	t	-0.84	0.69	0.09	1.69	0.17	0.03	-0.14
asleep surgery	B (SE)	0.15(0.13)	**0.52(0.23)**	-0.04(0.30)	-0.04(0.30)	-0.03(0.15)	0.15(0.23)	0.01(0.17)
	p	.27	**.03**	.89	.89	.82	.51	.94
	t	1.10	**2.22**	-0.13	-0.13	-0.23	0.67	-0.08
**Diagnosis astrocytoma (vs oligodendroglioma)**								
awake surgery	B (SE)	0.55(0.37)	-0.26(0.44)	0.16(0.52)	-0.21(0.63)	0.01(0.13)	0.20(0.40)	0.11(0.47)
	p	.14	.56	.76	.74	.98	.63	.82
	t	1.49	-0.59	0.31	-0.33	0.03	.49	0.23
asleep surgery	B (SE)	-0.08(0.35)	-0.94(0.44)	0.83(0.64)	0.83(0.65)	0.21(0.37)	-0.03(0.51)	0.69(0.50)
	p	.82	.04	.21	.21	.57	.95	.17
	t	-0.23	-2.13	1.27	1.29	0.58	-0.07	1.38
**Age (in years)**								
awake surgery	B (SE)	-0.00(0.01)	-0.02(0.01)	-0.01(0.01)	-0.05(0.02)	-0.01(0.01)	-0.01(0.01)	0.01(0.01)
	p	.78	.04	.61	.01	.22	.38	.62
	t	-0.28	-2.11	-0.52	-2.56	-1.23	-0.89	0.50
asleep surgery	B (SE)	-0.00(0.01)	0.00(0.01)	-0.01(0.01)	-0.01(0.01)	0.00(0.01)	-0.00(0.01)	-0.00(0.01)
	p	.82	.91	0.51	.51	.64	.77	.84
	t *p*	-0.23	0.12	-0.66	0.66	0.46	.30	-0.21
**Frontal location**								
awake surgery	B (SE)	0.11(0.28)	0.15(0.22)	-0.22(0.29)	-0.42(0.50)	-0.50(0.31)	0.34(0.25)	0.37(0.33)
	p	.71	.49	.46	.41	.11	.18	.28
	t	0.37	0.70	-0.75	-0.84	-1.63	1.35	1.09
asleep surgery	B (SE)	0.21(0.32)	0.37(0.23)	-0.68(0.48)	-0.68(0.48)	0.57(0.35)	-0.08(0.36)	-0.10(0.46)
	p	.52	.11	.16	.16	.11	.83	.94

	t	-0.64	1.63	-1.42	-1.42	1.62	-0.21	-0.20
**Temporal location**								
awake surgery	B (SE)	-0.39(0.8)	0.13(0.21)	0.24(0.28)	0.23(0.48)	-0.72(0.29)	-0.45(0.27)	-0.22(0.36)
	p	.16	.54	.40	.64	.02	.10	.55
	t	-1.43	0.52	0.85	0.47	-2.44	-1.69	-0.61
asleep surgery	B (SE)	-0.19(0.31)	0.39(0.22)	**-0.98(0.46)**	**-0.98(0.46)**	**0.97(0.35)**	0.13(0.34)	0.04(0.43)
	p	.53	.09	**.04**	**.04**	**.01**	.71	.93
	t	-0.64	1.74	**-2.11**	**-2.11**	**2.80**	0.37	0.08
**Time**								
awake surgery	B (SE)	0.02(0.13)	**-0.45(0.19)**	-0.10(0.23)	**-0.77(0.23)**	-0.09(0.13)	-0.11(0.14)	-0.03(0.12)
	p	.89	**.02**	.69	**<.01**	.49	.44	.83
	t	0.14	**-2.33**	-0.39	**-3.36**	-0.69	-0.78	-0.21
asleep surgery	B (SE)	-0.14(0.11)	**-0.52(0.18)**	-0.20(0.24)	-0.20(0.24)	0.16(0.13)	0.05(0.20)	-0.07(0.14)
	p	.19	**.01**	.41	.41	.22	.78	.63
	t	-1.31	**-2.80**	-0.82	-0.82	1.24	0.27	-0.48

*computerized test

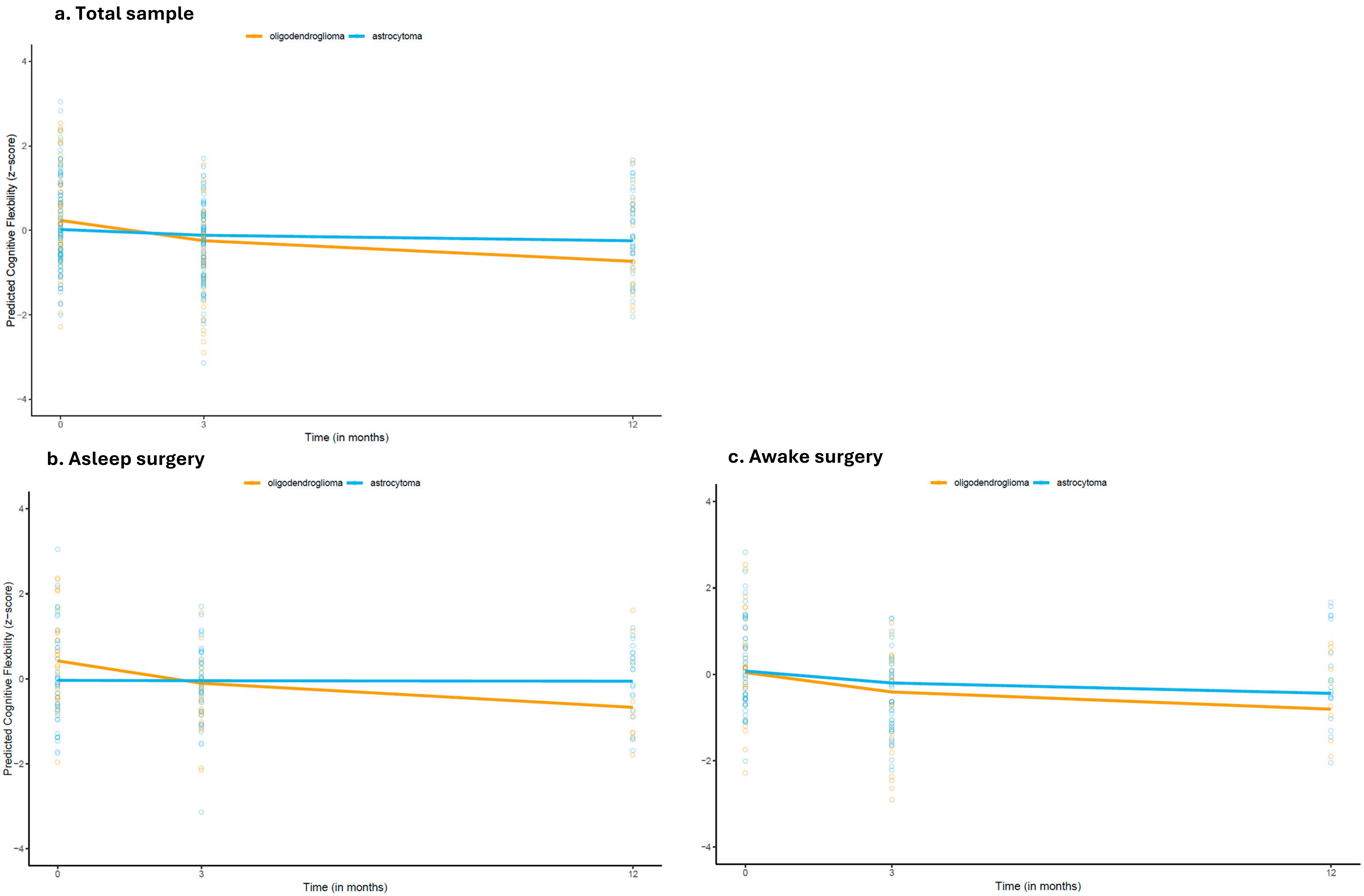

Supplementary figure 1. Estimated (mean) performances over time on the Shifting Attention test (measuring cognitive flexibility), in a) the entire sample, b) the sample undergoing awake surgery, and c) the sample undergoing asleep surgery. Estimated performances are based on the models that include diagnosis and clinical covariates, meaning performances are estimated while accounting for covariates. Colors indicate the diagnosis, with lines being the estimated group trajectory and circles being the estimated individual performances at each timepoint. In the asleep surgery group, oligodendroglioma patients performed worse on both the 3- and 12 month follow-up compared to their own preoperative baseline (p <.05, see main results). Differences between the two groups on the follow-up measurements were not significant (p >.05, see main results)

-- End of document --
